# Editorial: Rhizosphere microbiology: Toward a clean and healthy soil environment

**DOI:** 10.3389/fmicb.2022.991356

**Published:** 2022-08-02

**Authors:** Wanting Ling, Bin Ma, Wei Zhang

**Affiliations:** ^1^College of Resource and Environmental Sciences, Institute of Organic Contaminant Control and Soil Remediation, Nanjing Agricultural University, Nanjing, China; ^2^College of Environmental and Resource Sciences, Zhejiang University, Hangzhou, China; ^3^Department of Plant, Soil and Microbial Sciences, and Environmental Science and Policy Program, Michigan State University, East Lansing, MI, United States

**Keywords:** rhizosphere, microbiology, soil environment, root exudates, pollutant, fertility

The significance of soil to humanity and the important role it plays in ecosystem health and agriculture are conclusive. Soil is a living system that needs to be enriched through sustainable practices so as to support various activities that human societies depend upon it for. However, the burgeoning global human population has led to increased demand on food production despite a decrease in acreages of global croplands, consequently posing a risk to food security. Soil is also a repository of pollutants. Contamination of agricultural lands exacerbates the pressure of meeting the global food demand without jeopardizing the planetary ecosystems. As a living entity, soil must be restored in a sustainable way to achieve this need and soil rhizosphere should be a focal point of the restoration efforts. In arable agriculture integrated nutrient management is currently being focused on and the role of rhizosphere microorganisms needs to be further emphasized. Rhizosphere microorganisms can have a huge impact on soil health and agriculture as they carry out dual functions of promoting plant growth and remediating soil contamination. Plants also produce and secrete root exudates that regulate the diversity and activity of microorganisms on/around plant roots.

Given the rapidly evolving research in this area, the journal of *Frontiers in Microbiology* published a Research Topic titled “Rhizosphere Microbiology: Toward a Clean and Healthy Soil Environment”. This Research Topic aims to report the novel omics and high-throughput molecular approaches that either characterize novel rhizosphere bioresources and biomolecules or discover mechanisms of their actions in processes such as colonization, biofilm formation, rhizodeposition, and biomolecule production. The innovative approaches in rhizosphere microbiology will produce fundamental knowledge in rhizosphere microbial ecology and help develop practices that can improve food security, food safety, and environmental sustainability in polluted agricultural soils.

Rhizosphere microbiome has been recognized to play a defensive role in plant-pathogen interactions (Li, Wang et al.) and promote plant health and growth. Microbial communities in the rhizospheres of various plants have been extensively studied using the advanced biotechniques including computational biology, sequencing technology, and bioinformatics. For example, the taxonomic composition of rhizosphere bacterial communities of Ku Shen (*Sophora flavescens*) grown in various regions in China were determined using Illumina 16S amplicon sequencing and showed difference across geographic location and plant ages (Chen et al.). Geographic factors such as location, climate, and soil properties (pH, available N, and available P) are key determinants of the rhizosphere bacterial community, which is shown to be associated with the accumulation of alkaloids in Ku Shen plants. Ahmed et al. studied the diversity and structure of microbial communities in hemp roots and rhizospheres using Illumina MiSeq amplicon sequencing of bacterial 16S rDNA and fungal ITS and reported the dominance of *Planctobacteria* and *Ascomycota* among hemp-associated microorganisms. The microbial communities in rhizosphere of Oil tea (*Camellia spp*.) and roselle were reported (Li, Zhang et al.; Wang et al.). The rhizosphere microbiome of disease-resistant *Camellia yuhsienensis* has higher richness and diversity, more symbiotic fungal communities, and fewer pathogens than that of high-yield, disease-susceptible *Camellia oleifera* (Li, Zhang et al.). The microbial compositions in the roselle rhizosphere was studied using fungal ITS and bacterial 16S rRNA amplicon sequencing (Wang et al.). *Fusarium* species were identified as the main pathogen targets to roselle and *F. solani* was found as the pathogen responsible for the roselle wilt disease (Wang et al.). Zhang J. et al. investigated the taxonomy and co-occurrence relationships of protists in the rhizosphere of soybean fields from six ecological regions of China and found more extensive and complex protist network in the bulk soil than in the rhizosphere, probably due to less overlaps and interactions of niches in the rhizosphere.

Rhizosphere microbial communities are significantly affected by the cropping management. Gu et al. found that intermittent deep tillage altered rhizosphere bacterial communities and their function profiles, and enhanced plant physiology and disease resistance, resulting in increased crop yield. In a study on a traditional intercropping system of maize and common bean (milpa) in Mesoamerica, Aguirre-Noyola et al. elucidated the transcriptomic responses of *Rhizobium phaseoli* Ch24-10 to the root exudates of maize and bean grown in either monoculture or the milpa system, which demonstrated the ability of rhizobium phaseoli to colonize maize and common bean in the intercropping system. Continuous cropping can negatively influence soil quality and thus the yield and quality of crops. The evolutionary trend of soil microbiomes as driven by continuous cropping was reviewed by Chen et al. The accumulation of autotoxic substances such as phenolic acids from the continuous cropping could degrade soil quality, and phenolic acids were found toxic to *Panax notoginseng* by promoting the growth of bacterial pathogens and disturbing rhizosphere microbiome (Bao et al.). Compost application could provide plants with microbial inoculants and Wang N. et al. found that the rhizosphere microbiomes of tomato, pepper, or maize receiving compost application were collectively determined by compost type and plant species. Deng et al. found that the rhizosphere microbiome responds differently to the different level of P addition. Similarly, Zhang S. et al. reported that long-term fertilization increased the spore density of arbuscular mycorrhizal fungi (AMF) but decreased the AMF diversity. Glomus was identified as the dominant genus in the soil and root samples.

Novel bioresources such as beneficial microorganisms and biomolecules could be useful to safeguard the soil environment. Field experiments revealed that Aspergillus and Bacillus inoculation could increase cotton yield at even lower inoculant concentrations (Escobar Diaz et al.). Phosphate-solubilizing bacteria (PSB) is important to P cycling and increase P availability in P-deficient soils. He and Wan reported that the soybean growth was significantly increased by the inoculation of PSB *Acinetobacter pittii* gp-1, likely due to enhanced P availability, because P-cycling-related gene abundance (i.e., *phoD, bpp, gcd*, and *pstS*) and enzyme activities (e.g., phosphotransferase) in the rhizosphere were increased. Yahya et al. isolated seven PSBs, which had efficient *in vitro* P-solubilizing activity. The PSBs can increase P content in wheat by modulating morphophysiology and exudation of roots and reduce oxidative stress at P-deficit conditions. Liu et al. isolated *Serratia marcescens* strain JW-CZ2 from the rhizosphere soils of tea plants. This strain has a high 1-aminocyclopropane-1-carboxylic acid (ACC) deaminase activity and could solubilize inorganic phosphate, suppress the growth of fungal pathogens, and generate indole acetic acid and siderophore, which leads to enhanced tea plant growth. When a plant growth-promoting bacterium *Bacillus velezensis* JC-K3 was inoculated into the rhizosphere of wheat plants, wheat growth was enhanced and the microbial abundance and diversity within wheat plants and in the rhizosphere were significantly changed so as to reduce plant damage to salt stress (Ji et al.). Abdelrahman et al. evaluated the potential of Actionmycetes for biological control to protect plants against Phytophthora infestans and found promising results based on 175 Actinomycete isolates from the Sudan's soils. Chemotaxis is important to bacteria adaptation and survival in soil environment and Liu et al. reported that hypoxia condition and cellular energy-status affect the localization of protein CheZ that regulates the chemotaxis of *Azorhizobium caulinodans* ORS571.

The interaction of pollutants with microorganisms in the rhizosphere were also addressed. Heavy metals, organic chemical pollutants, and antibiotic resistance genes (ARGs) are of great concerns. González et al. studied the rhizosphere microbial communities in the Hg-contaminated soils and found that plant roots select the rhizosphere microbial community with Proteobacteria as the most abundant phylum. Zhao et al. isolated a phenanthrene-degrading bacterium *Diaphoro bacter sp*. Phe15 from root surfaces in fields contaminated with polycyclic aromatic hydrocarbons (PAHs) and further demonstrated that the inoculation of Phe15 *via* roots or seeds dramatically decreased the phenanthrene concentrations in white clover (*Trifolium repens* L.). Additionally, Zhou et al. characterized the different molecular size fractions of glomalin-related soil proteins (GRSP) and revealed the interaction of various GRSP with PAHs. The phenolic group of GRSP is positively correlated with the binding of GRSP with phenanthrene. Finally, Wang T. et al. found that nitrogen application influenced the abundance of ARGs but not their distribution in leaves or roots for a soil–Chinese cabbage system.

Altogether this Research Topic published 24 papers contributed by authors from around the globe. Their high-quality contribution is greatly appreciated. We like to thank the outstanding work of all the referees. Finally, we thank the Editor-in-Chiefs, Research Topics Editors, and the Journal Managers for encouragement and support throughout the entire editing and publishing process of this Research Topic.

## Author contributions

All authors listed have made a substantial, direct, and intellectual contribution to the work and approved it for publication.

## Funding

This work was financially supported by the National Natural Science Foundation of China (41977121 and 42177016) and the Jiangsu Agricultural Science and Technology Innovation Fund (CX[21]2033).

## Conflict of interest

The authors declare that the research was conducted in the absence of any commercial or financial relationships that could be construed as a potential conflict of interest.

## Publisher's note

All claims expressed in this article are solely those of the authors and do not necessarily represent those of their affiliated organizations, or those of the publisher, the editors and the reviewers. Any product that may be evaluated in this article, or claim that may be made by its manufacturer, is not guaranteed or endorsed by the publisher.

